# Supplementary material for: Triethylamine vapor-induced cyclization reaction in cocrystals leading to cocrystal-to-polycrystal transformation
Source: Nat Commun. 2025 Jul 11;16:6441. doi: 10.1038/s41467-025-61782-2 (PMC12254319; doi:10.1038/s41467-025-61782-2)
Supplement: Supplementary file 1 — Supplementary Information [file 41467_2025_61782_MOESM1_ESM.pdf]

Supplementary Information

Triethylamine Vapor-Induced Cyclization Reaction in Cocrystals Leading to Cocrystal-to-Polycrystal Transformation

Ling Zhu[1], Xiaoli Zhao[1], Yanfei Niu[1], Lianrui Hu[1*], Weitao Dou[1], Hai-Bo Yang[1,2], Lin Xu[1,2*], Ben Zhong Tang[3*]

[1]State Key Laboratory of Petroleum Molecular & Process Engineering, Shanghai Key Laboratory of Green Chemistry and Chemical Processes, School of Chemistry and Molecular Engineering, East China Normal University, 3663 N. Zhongshan Road, Shanghai 200062, China

[2]Hainan Institute of East China Normal University, Sanya 572025, China

[3]School of Science and Engineering, Shenzhen Institute of Molecular Aggregate Science and Technology, The Chinese University of Hong Kong, Shenzhen (CUHK-Shenzhen), 2001 Longxiang Boulevard, Longgang District, Shenzhen, Guangdong 518172, China

Catalogue

Section A. Supplementary methods 3
1.1 The synthetic protocol of AIC and AIO 3
1.2 Density functional theory calculation details 3
Section B. Supplementary the basic properties of AOTC 4
2.1 Crystallographic data for AOTC. 4
2.2 Characterization of AOTC. 5
2.3 Theoretical calculations for AOTC. 7
Section C. Cocrystal-to-polycrystal transformation 10
3.1 Experimental details for cocrystal transformation with NEt_3 10
3.2 Crystallographic data for ACTC and AIC. 11
3.3 Supplementary materials for the cocrystal-to-polycrystal transformation 14
Section D. Characterization of new compounds 21
Section E. Supplementary X-ray crystallographic data. 23
Reference 25

Section A. Supplementary methods

1.1 The synthetic protocol of AIC and AIO

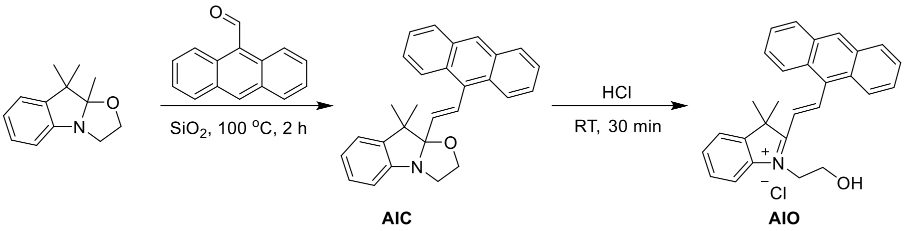

1.2 Density functional theory calculation details

All computations were conducted using Gaussian 16[1], employing density functional theory (DFT). The compounds underwent full optimization with the CAM-B3LYP[2] functional and a 6-31+G* basis set. To account for long-range inter/intramolecular interactions, Grimme's DFT-D3 correction was applied to consider London-dispersion effects[3,4]. Analytical frequency calculations were performed at the same level of theory to determine whether the calculated structure was intermediate (no virtual frequency) or the transition state (only one virtual frequency). Free energies were calculated at 298.15 K, 1 atm (unit: kcal/mol). The solvent effect was modelled in these single point calculations by employing SMD continuum solvation model, with triethylamine as the solvent for cyclization reaction. The reported free energies in this work were based on the electronic energy of solvation single point calculations, including the Gibbs free energy thermal correction obtained from vibrational analysis in gas phase, as well as entropy correction[5]. Entropy correction has been proved to be very robust and given reasonable results in a lot of theoretical calculations, has been used to reduce the overestimation of entropy[6,7]. All geometric structures are visualized using CYLview[8]. Frontier molecular orbitals (FMO) were visualized using the IQmol molecular viewer package (Isovalue: 0.5). Hirshfeld surface analysis through Crystal Explorer was employed to analyze intermolecular interactions in crystals. Additionally, the independent gradient model based on Hirshfeld partition (IGMH) analyses based on optimized structures and the energy decomposition analysis based on the force field (EDA-FF) were conducted using the independent gradient model in Multiwfn 3.8[9] and visualized using VMD software (version 1.9.3)[10].

Section B. Supplementary the basic properties of AOTC

2.1 Crystallographic data for AOTC.

Supplementary Table 1 | Crystallographic Data for AOTC.

Molecular crystals	AOTC
Empirical formula	$C_{33}H_{27}ClN_3O$
Formula weight	517.02
Temperature / K	172.99(10)
Crystal system	triclinic
Space group	$P\bar{1}$
a / Å	9.9613(10)
b / Å	10.1534(11)
c / Å	13.7735(7)
α / °	93.903(6)
β / °	91.619(6)
γ / °	108.571(9)
V / Å^3	1315.6(2)
Z	2
ρ_{calcd} / g cm^{-3}	1.305
μ / mm^{-1}	1.527
F (000)	542.0
Goodness-of-fit on F^2	1.008
Final R indexes [I >= 2σ (I)]	R_1 = 0.0669, wR_2 = 0.1736
Final R indexes [all data]	R_1 = 0.0789, wR_2 = 0.1842
CCDC No.	2343127

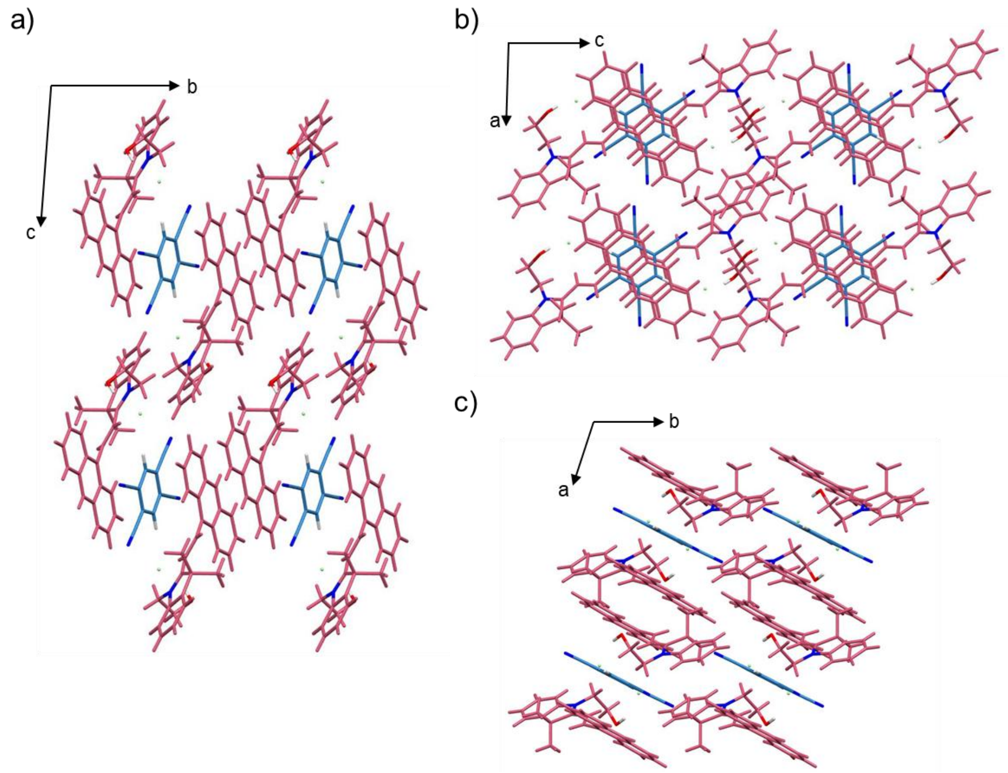

Supplementary Fig. 1 | Packing modes of AOTC crystal. Molecular packing modes of AOTC crystal perpendicular to the (a) a-axis, (b) b-axis, and (c) c-axis.

2.2 Characterization of AOTC.

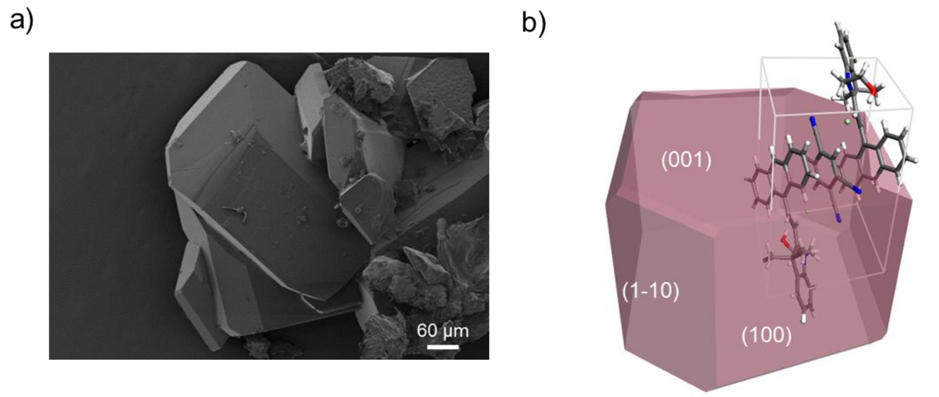

Supplementary Fig. 2 | Morphology of AOTC cocrystal. a) SEM images of AOTC cocrystals. b) The simulated morphology of AOTC cocrystal based on growth morphology method.

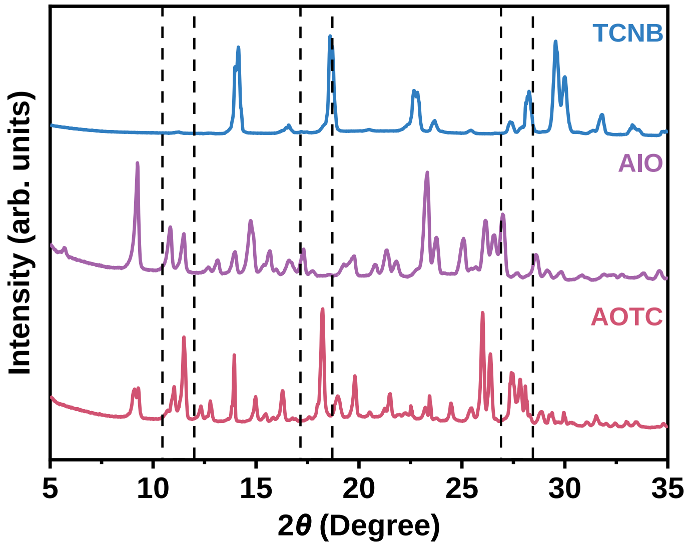

Supplementary Fig. 3 | PXRD analyses. PXRD spectrum of AOTC, AIO and TCNB.

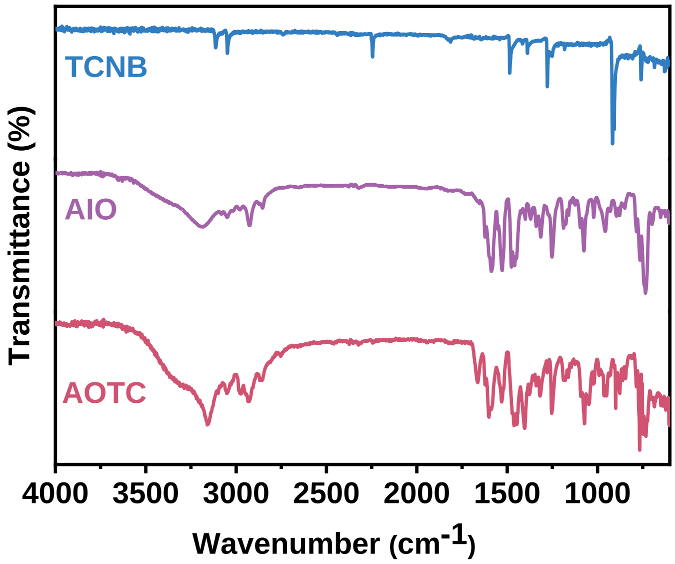

Supplementary Fig. 4 | FTIR analyses. FTIR spectra of AOTC, AIO and TCNB.

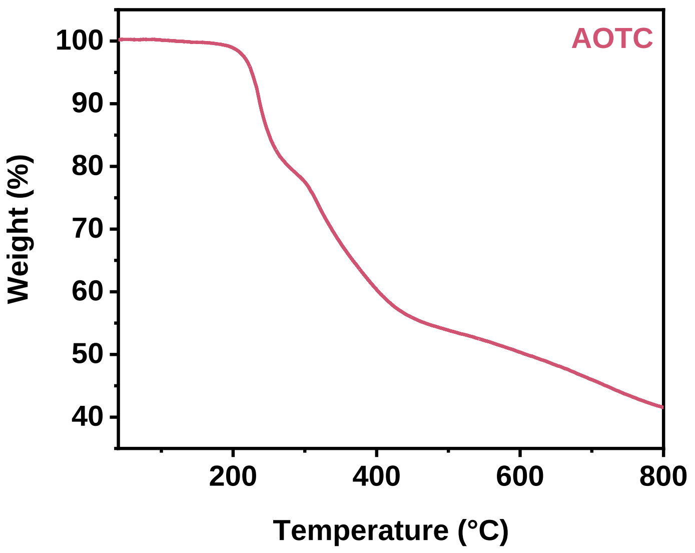

Supplementary Fig. 5 | TGA analysis. TGA thermogram of AOTC cocrystal recorded under nitrogen at a heating rate of 10 °C·min^{-1}.

2.3 Theoretical calculations for AOTC.

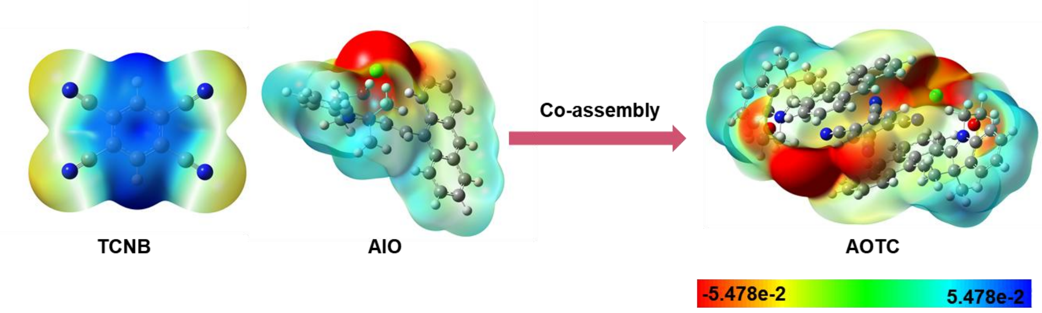

Supplementary Fig. 6 | Electronic static potential of TCNB, AIO and AOTC. Electronic static potential mapped on the isosurface of electronic density, based on their optimized ground-state geometries. A negative electrostatic potential (red) represents a high electronic density, while a positive one (blue) corresponds to a low electronic density.

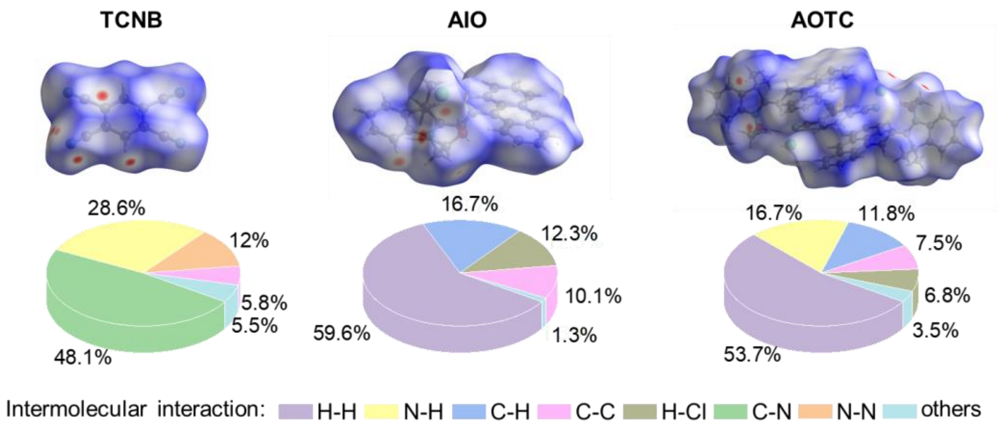

Supplementary Fig. 7 | Hirshfeld surface analyses. Hirshfeld surface analysis plots (mapped over dnorm) of TCNB, AIO and AOTC.

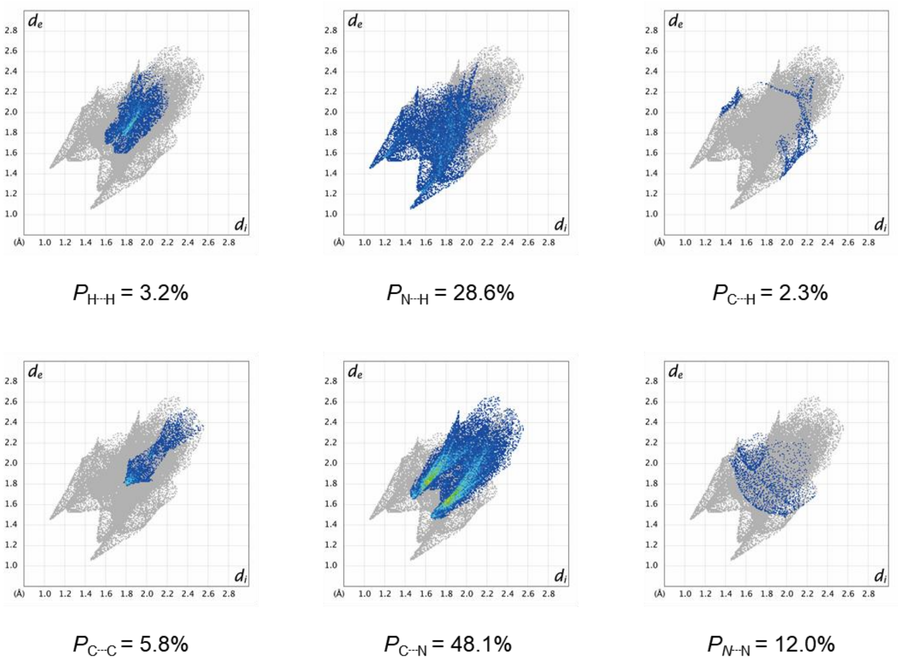

Supplementary Fig. 8 | Hirshfeld surfaces and decomposed fingerprint plots of TCNB. Full fingerprints appeared as grey shadows underneath decomposed plots, and selected intermolecular interactions were shown as a blue shadow. The proportions (*P*) of different kinds of intermolecular interactions to total intermolecular interaction were also indicated.

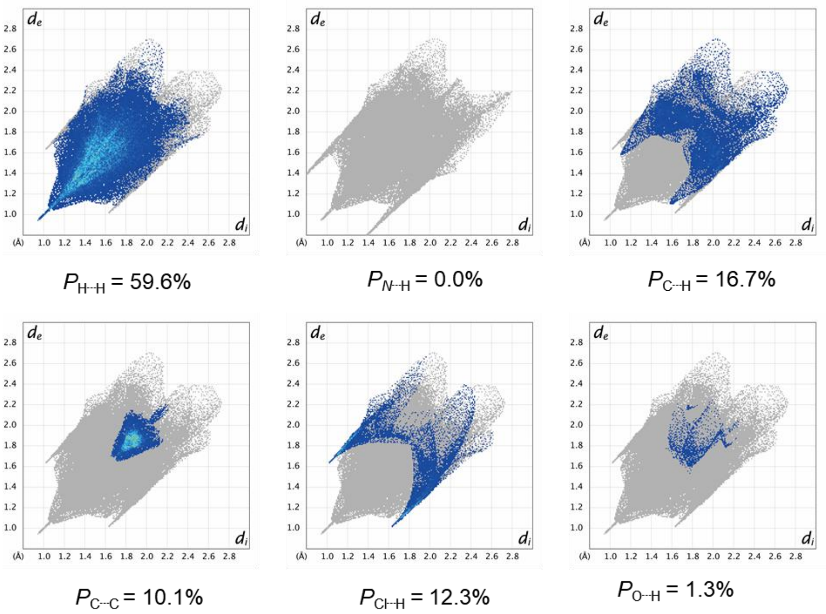

Supplementary Fig. 9 | Hirshfeld surfaces and decomposed fingerprint plots of AIO. Full fingerprints appeared as grey shadows underneath decomposed plots, and

selected intermolecular interactions were shown as a blue shadow. The proportions (P) of different kinds of intermolecular interactions to total intermolecular interaction were also indicated.

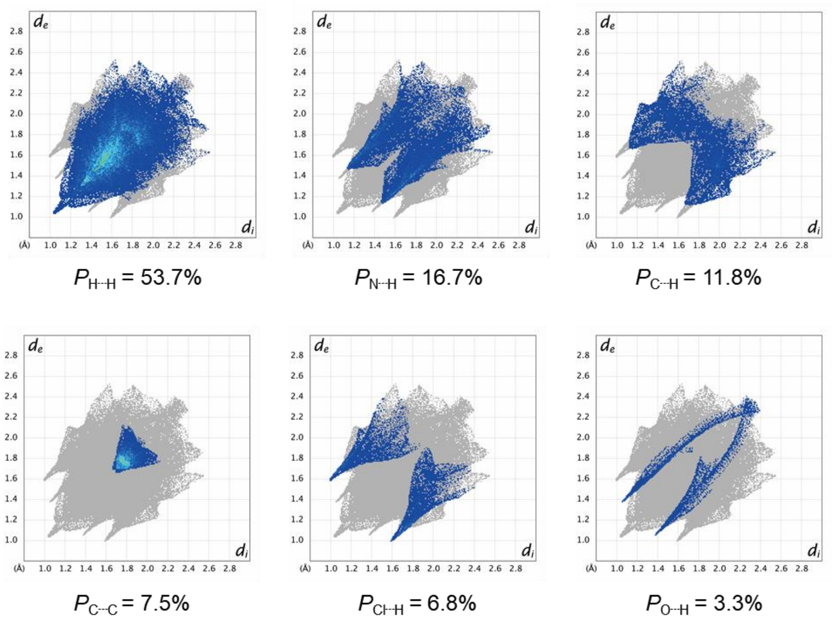

Supplementary Fig. 10 | Hirshfeld surfaces and decomposed fingerprint plots of AOTC. Full fingerprints appeared as grey shadows underneath decomposed plots, and selected intermolecular interactions were shown as a blue shadow. The proportions (P) of different kinds of intermolecular interactions to total intermolecular interaction were also indicated.

Section C. Cocrystal-to-polycrystal transformation

3.1 Experimental details for cocrystal transformation with NEt_3

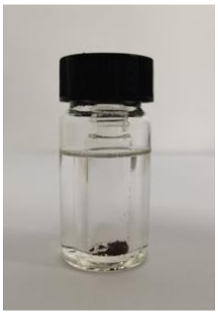

Supplementary Fig. 11 | Experiment of vapor-induced transformation. Schematic diagrams of the AOTC upon exposure to NEt_3 vapor.

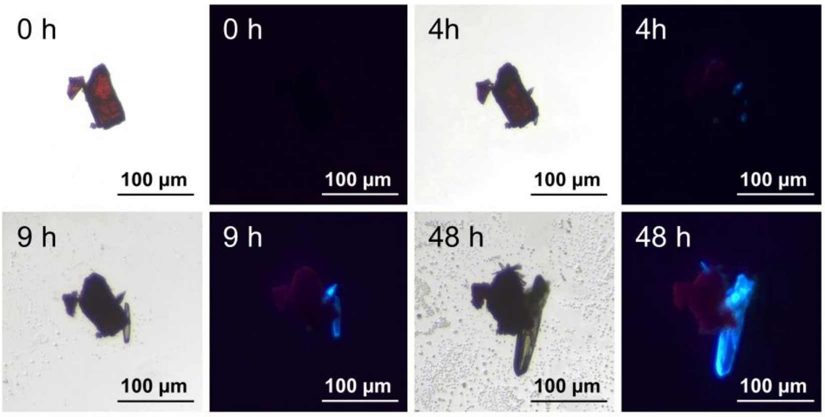

Supplementary Fig. 12 | The photographs of AOTC cocrystals exposed to NEt_3 vapor for various durations. All photos were taken under room light (left) and a 365 nm UV lamp (right). These images demonstrate the transformation from AOTC cocrystal to AIC crystal (bule-emissive) and ACTC cocrystal (red-emissive).

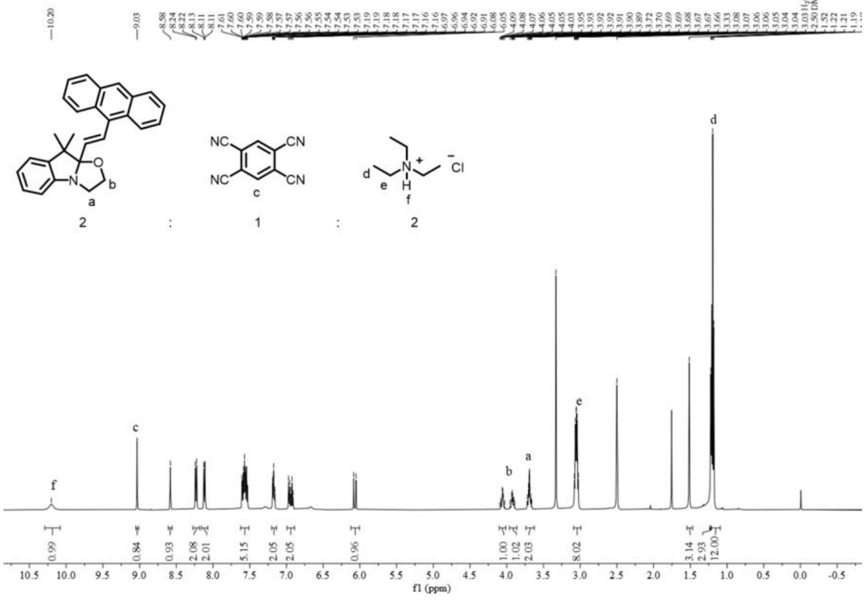

Supplementary Fig. 13 | The NMR analysis of AOTC+NEt$_3$. The ^{1}H NMR spectrum of the AOTC+NEt$_3$ crystals (DMSO-d_6, 500 MHz). The quantity of triethylamines is slightly higher than the theoretical expectations, due to trace amounts of free triethylamine molecules remaining in the sample.

3.2 Crystallographic data for ACTC and AIC.

Supplementary Table 2 | Crystallographic Data for ACTC and AIC.

Molecular crystals	ACTC	AIC
Empirical formula	$C_{38}H_{27}N_5O$	$C_{28}H_{25}NO$
Formula weight	569.64	391.49
Temperature / K	193.00	173.00(10)
Crystal system	orthorhombic	orthorhombic
Space group	$Pca2_1$	$Pca2_1$
a / Å	42.432(4)	18.6301(12)
b / Å	7.4563(5)	7.3457(7)
c / Å	9.3936(8)	30.314(2)
α / °	90	90
β / °	90	90
γ / °	90	90
V / Å^3	2972.0(4)	4148.6(6)
Z	4	8
ρ_{calcd} / g cm^{-3}	1.273	1.254
μ / mm^{-1}	0.619	0.581
F (000)	1192.0	1664.0
Goodness-of-fit on F^2	0.959	0.980
Final R indexes [I >= 2σ (I)]	R_1 = 0.0763, wR_2 = 0.1931	R_1 = 0.1130, wR_2 = 0.2564
Final R indexes [all data]	R_1 = 0.1140, wR_2 = 0.2281	R_1 = 0.1703, wR_2 = 0.3027
CCDC No.	2343126	2343124

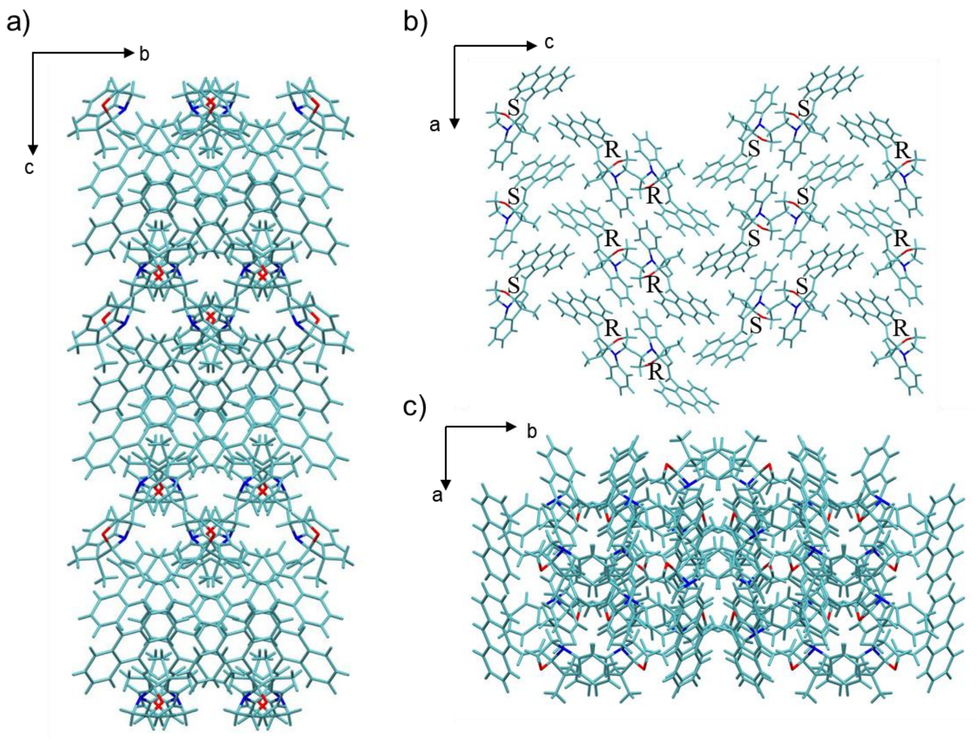

Supplementary Fig. 14 | Packing modes of AIC crystal. Molecular packing modes of AIC crystal perpendicular to the a) a-axis, b) b-axis, and c) c-axis.

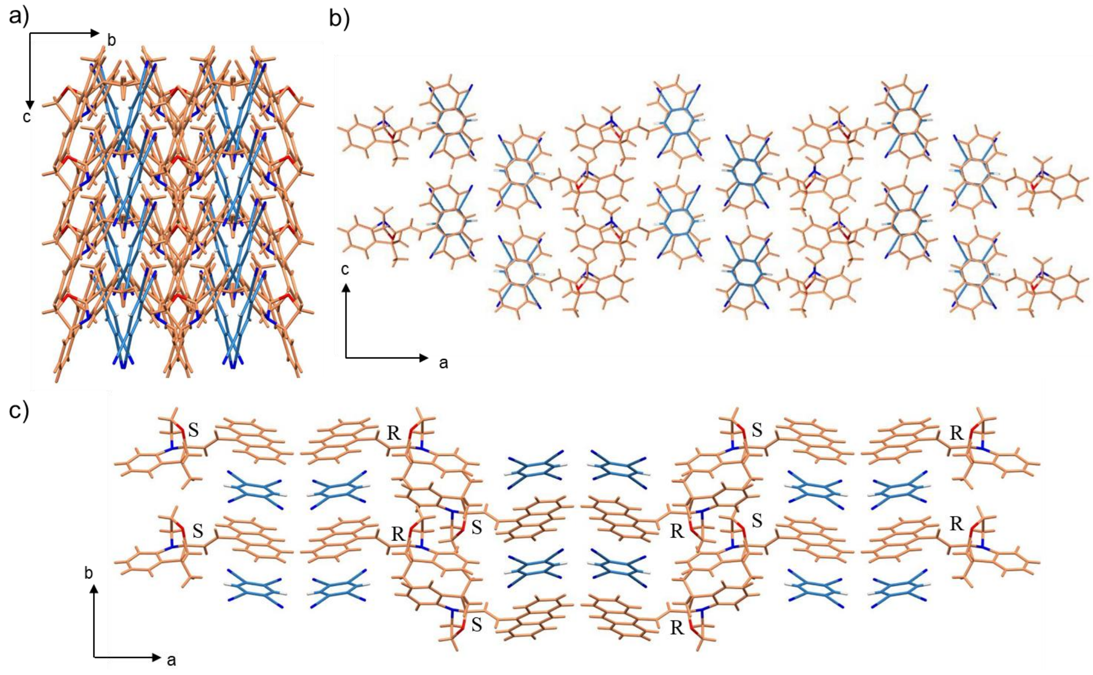

Supplementary Fig. 15 | Packing modes of ACTC cocrystal. Molecular packing modes of ACTC crystal perpendicular to the a) a-axis, b) b-axis, and c) c-axis.

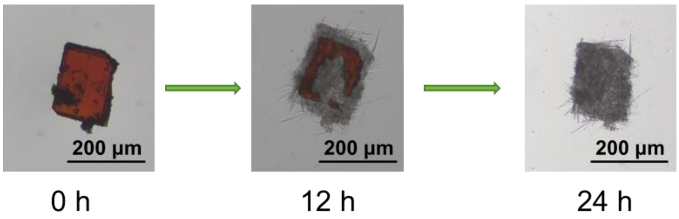

Supplementary Fig. 16 | Transformation of AOTC cocrystal in liquid triethylamine. Optical microscope images of AOTC cocrystal immersed in liquid triethylamine at different time intervals show that AIC dissolved, while residual TCNB crystals remained. This is attributed to the higher solubility of AIC compared to TCNB in triethylamine.

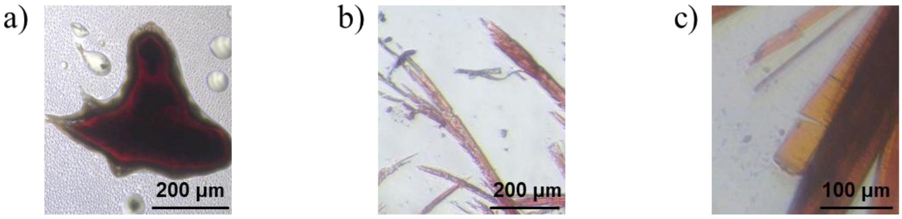

Supplementary Fig. 17 | Optical microscope images of the ACTC cocrystal after exposure to different acids. Images show the ACTC cocrystal after treatment with a) CF_3COOH, b) AcOH, and c) HCl.

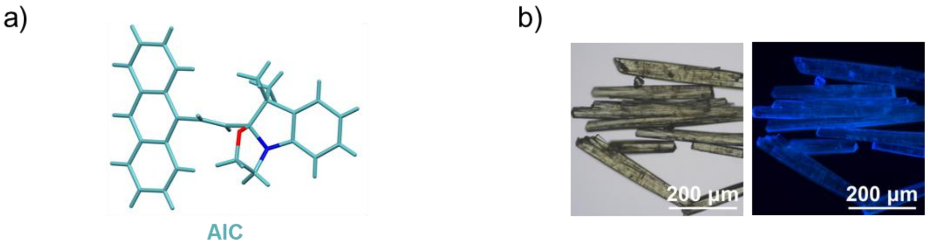

Supplementary Fig. 18 | Preparation of AIC crystal. a) Molecular structures of AIC. b) The photographs of AIC crystals taken under room light (left) and a 365 nm UV lamp (right).

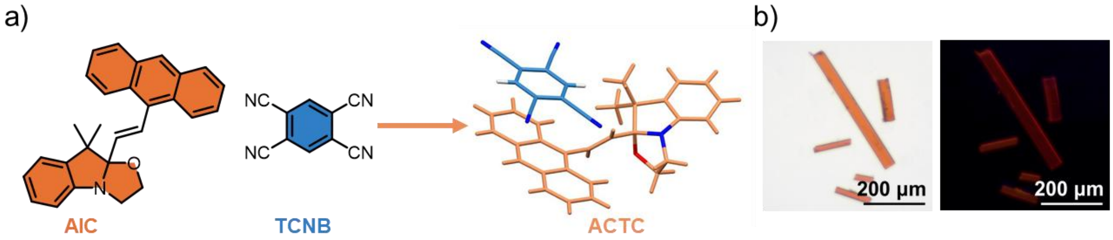

Supplementary Fig. 19 | Preparation of ACTC cocrystal. a) Molecular structures of ACTC. b) The photographs of ACTC crystals taken under room light (left) and a 365 nm UV lamp (right).

3.3 Supplementary materials for the cocrystal-to-polycrystal transformation

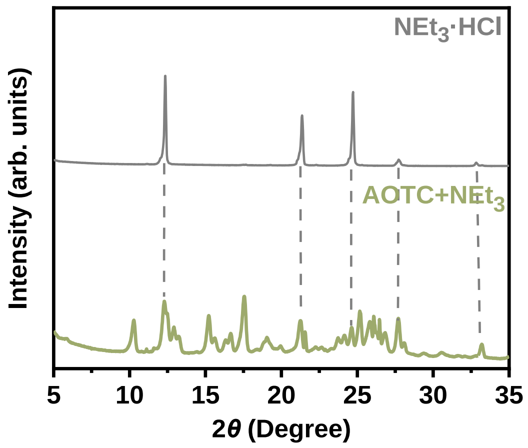

Supplementary Fig. 20 | PXRD analyses of AOTC+NEt_3. PXRD patterns of NEt_3·HCl and AOTC+NEt_3 demonstrate the presence of NEt_3·HCl solid in AOTC+NEt_3.

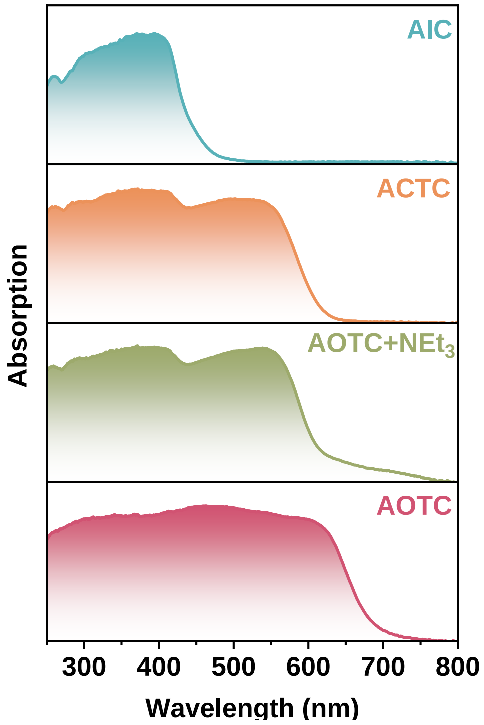

Supplementary Fig. 21 | UV-Vis absorption spectra. Solid-state UV-Vis absorption spectra of AIC, ACTC, AOTC+NEt_3, and AOTC.

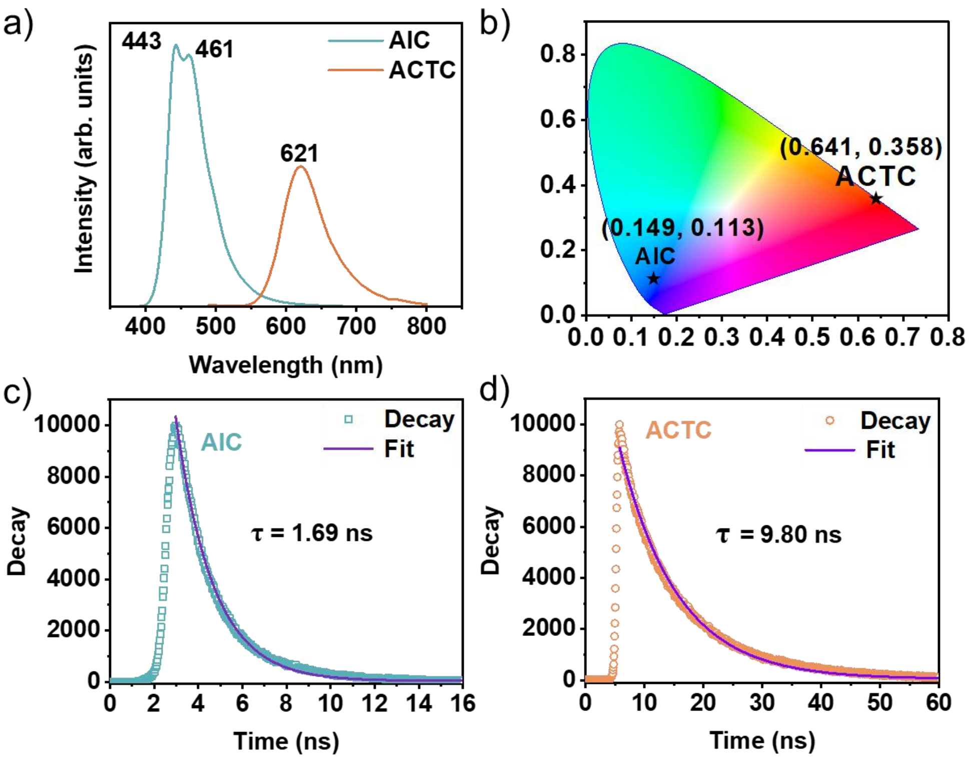

Supplementary Fig. 22 | Fluorescence spectra analyses of AIC and ACTC. a) Solid-state fluorescence spectra of AIC and ACTC. b) CIE chromaticity diagram of AIC and ACTC. The fluorescence decay curves for the c) AIC crystal (λ = 375 nm) and d) ACTC cocrystal (λ = 375 nm).

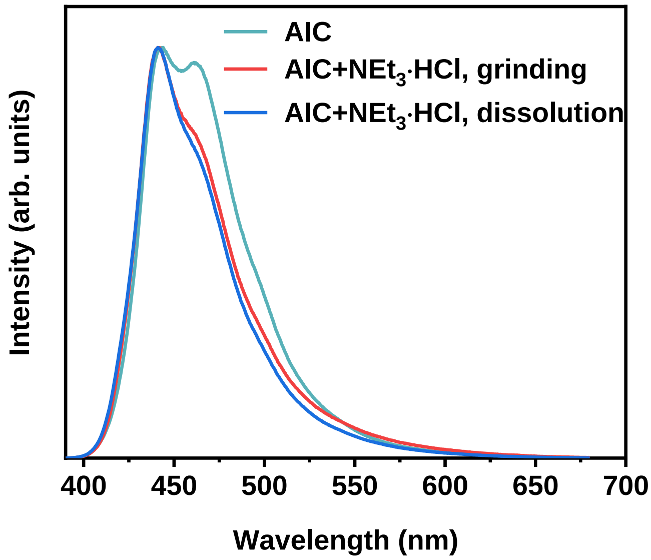

Supplementary Fig. 23 | Solid-state fluorescence spectra of AIC and AIC+NEt_3·HCl prepared via different mixing methods. The experimental results show that the solid-state fluorescence emission of AIC changes upon mixing with solid NEt_3·HCl. This fluorescence changes more pronounced when the mixing method

change from simple mechanical grinding to dissolution mixing. Therefore, we speculate that the slight difference in fluorescence between AOTC+NEt_3 and pure AIC may be attributed to the presence of $NEt_3{\cdot}HCl$.

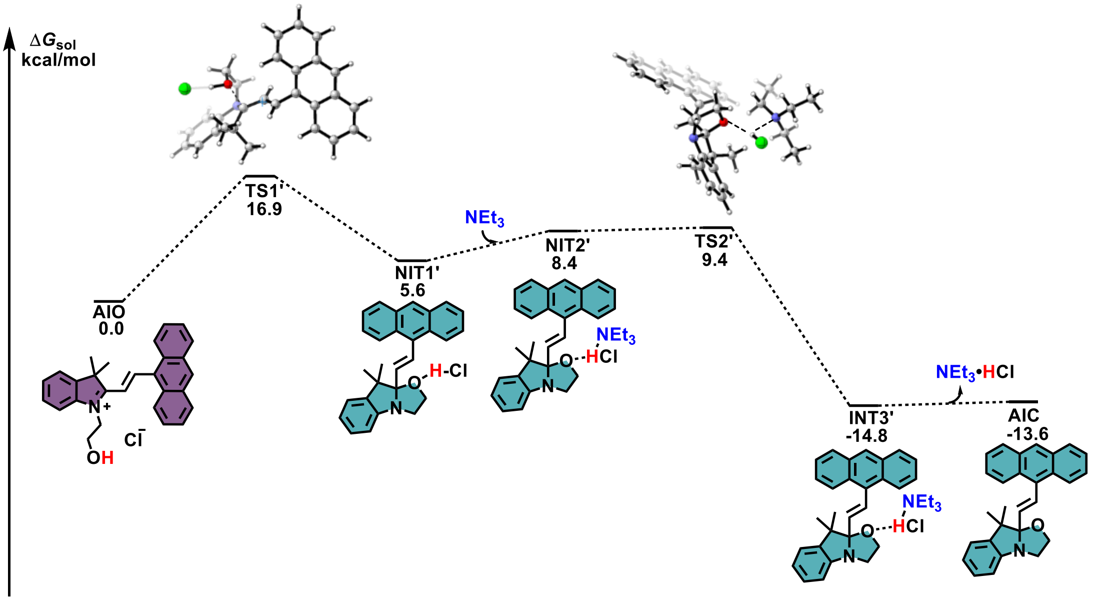

Supplementary Fig. 24 | Free energy changes of AIO cyclization reaction. DFT computed free energy changes for the cyclization reaction of AIO at the CAM-B3LYP/6-31+G* level with SMD solvent model (triethylamine).

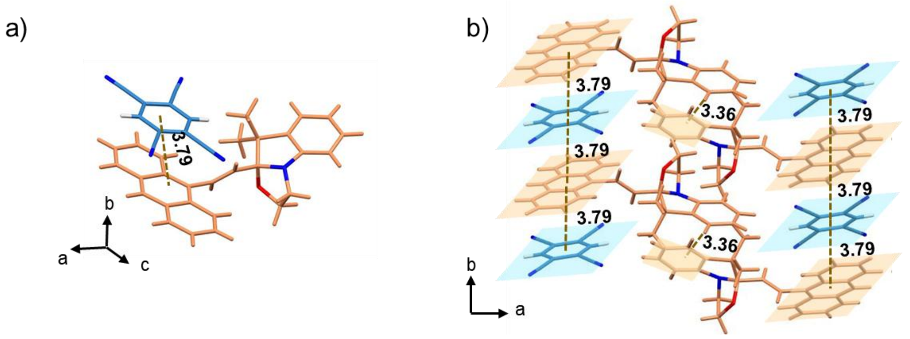

Supplementary Fig. 25 | Solid-state superstructure of the ACTC cocrystal. a) The face-to-face packing between AIC and TCNB. b) The π···π bonds of ACTC.

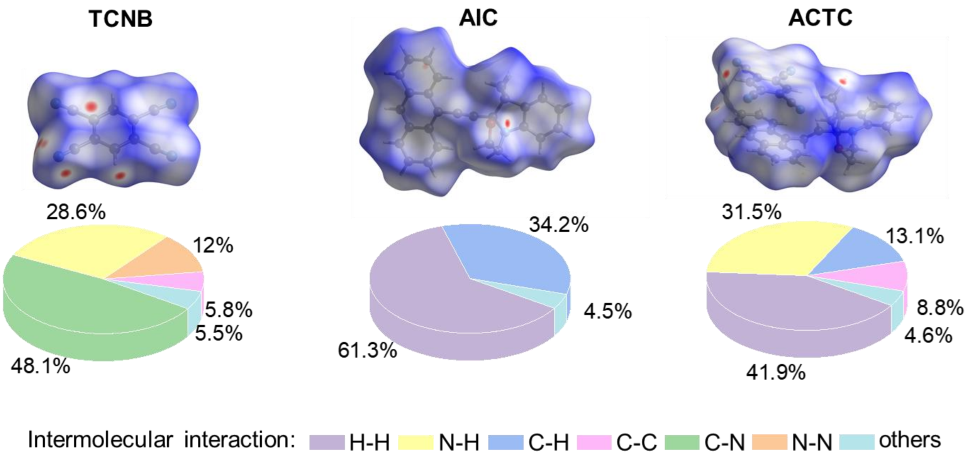

Supplementary Fig. 26 | Hirshfeld surface analyses. Hirshfeld surface analysis plots (mapped over dnorm) of TCNB, AIC and ACTC.

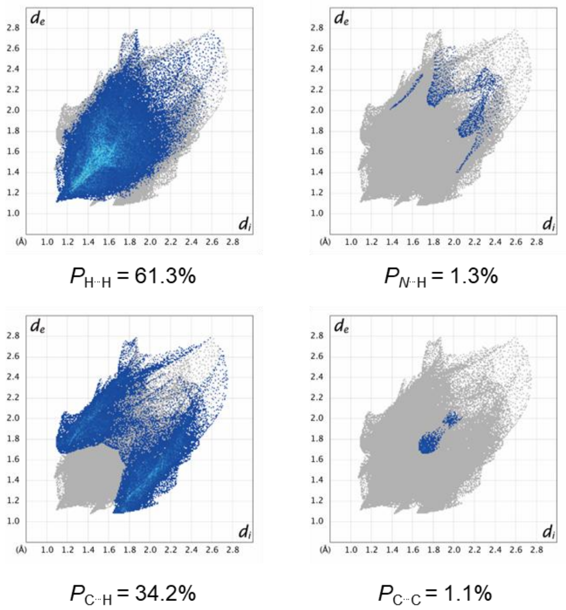

Supplementary Fig. 27 | Hirshfeld surfaces and decomposed fingerprint plots of AIC. Full fingerprints appeared as grey shadows underneath decomposed plots, and selected intermolecular interactions were shown as a blue shadow. The proportions (*P*) of different kinds of intermolecular interactions to total intermolecular interaction were also indicated.

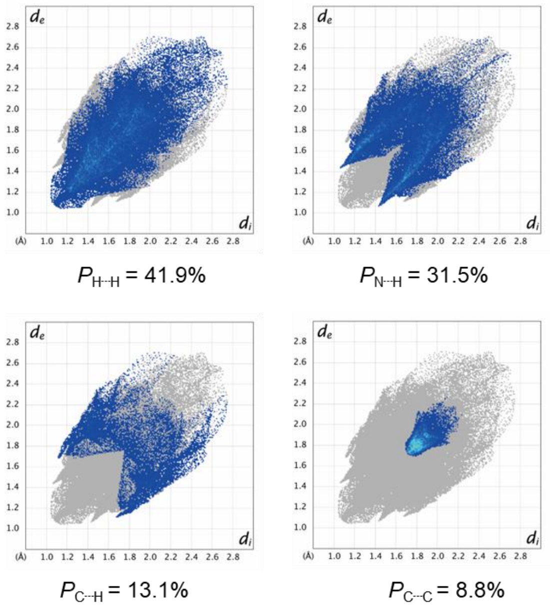

Supplementary Fig. 28 | Hirshfeld surfaces and decomposed fingerprint plots of ACTC. Full fingerprints appeared as grey shadows underneath decomposed plots, and selected intermolecular interactions were shown as a blue shadow. The proportions (*P*) of different kinds of intermolecular interactions to total intermolecular interaction were also indicated.

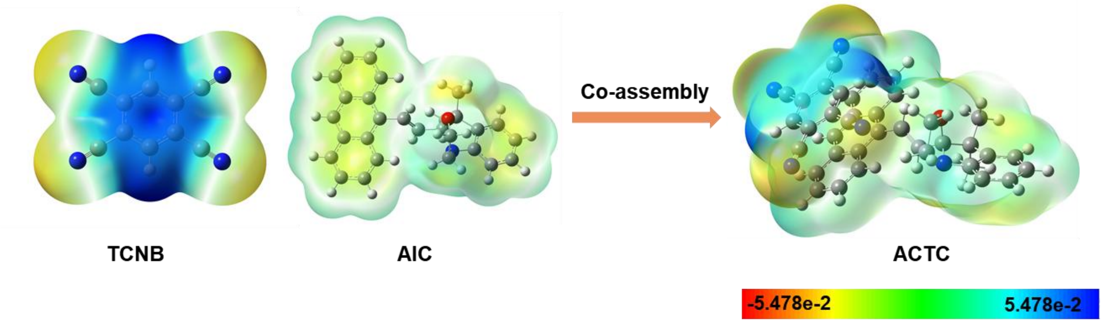

Supplementary Fig. 29 | Electronic static potential of TCNB, AIC and ACTC. Electronic static potential mapped on the isosurface of electronic density, based on their optimized ground-state geometries. A negative electrostatic potential (red) represents a high electronic density, while a positive one (blue) corresponds to a low electronic density.

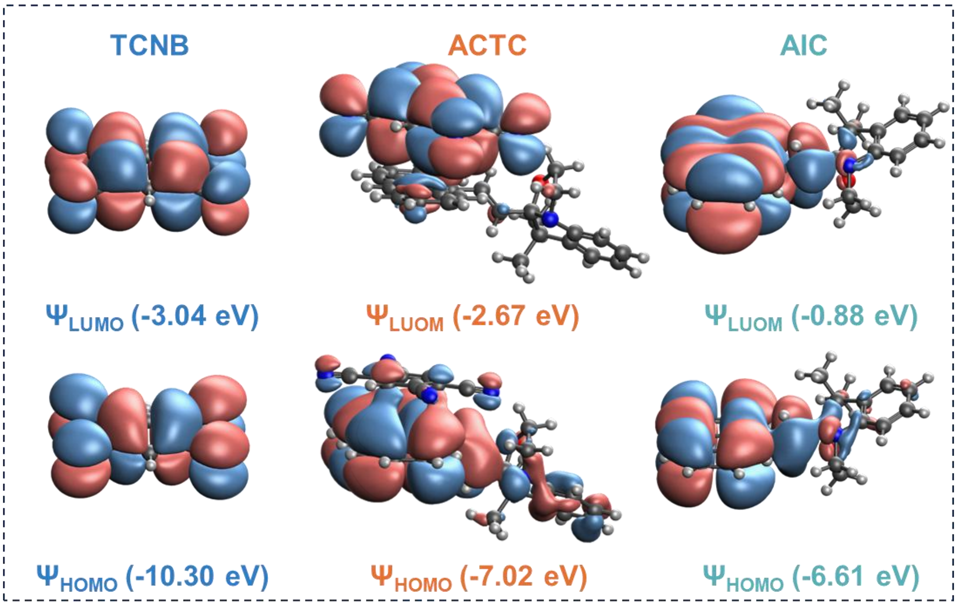

Supplementary Fig. 30 | Frontier molecular orbitals analysis. The HOMOs and LUMOs of TCNB, ACTC and AIC.

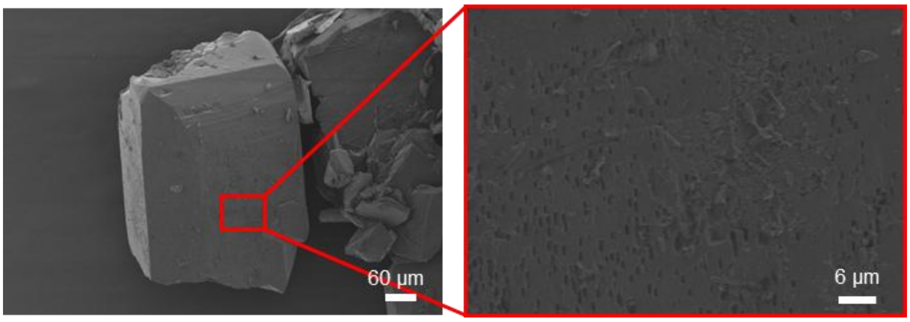

Supplementary Fig. 31 | SEM analysis. SEM images of AOTC cocrystal after exposure to NEt_3 vapor for about 2 hours (left) and partial enlarged detail (right).

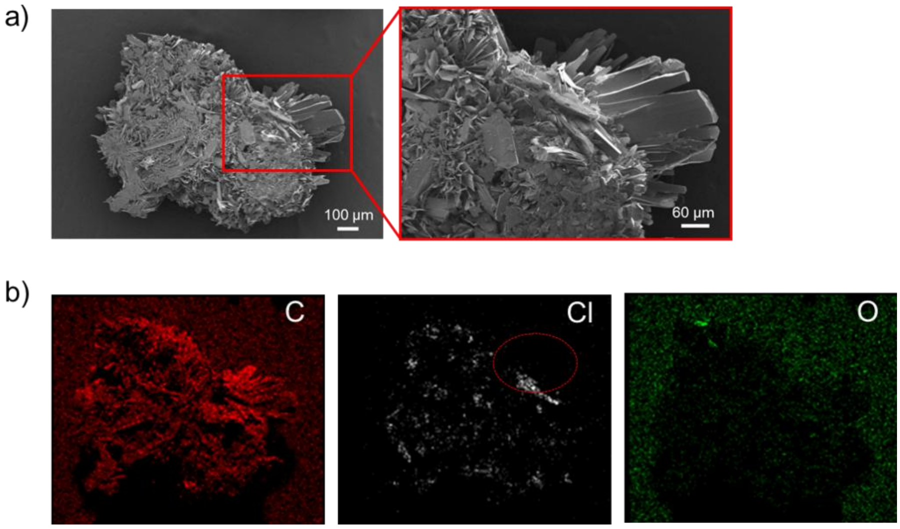

Supplementary Fig. 32 | SEM mages of AOTC+NEt3. a) SEM images of AOTC+NEt_3 (left) and partial enlarged detail (right). The new AIC crystals and ACTC cocrystals are distributed on the surface of AOTC crystals. b) SEM-EDS Maps of AOTC+NEt_3 cocrystals showing the distribution of the component elements (C, O, Cl). There is no Cl element in the new crystal part (circled by the red dotted line), indicating the closed form Box in the new crystals.

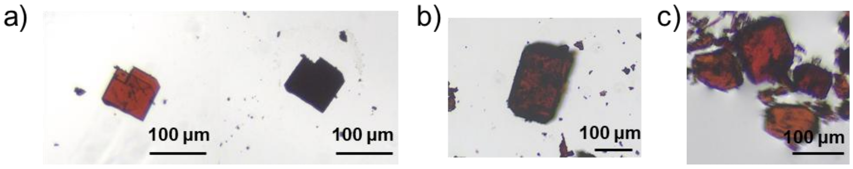

Supplementary Fig. 33 | Optical microscope images of AOTC cocrystal exposed to different bases. a) Images of the AOTC cocrystal before and after exposure to NH_3 vapor. b) Images of the AOTC cocrystal after exposure to *N, N*-Diisopropylethylamine vapor. c) Images of the AOTC cocrystal after immersion in a sodium carbonate solution.

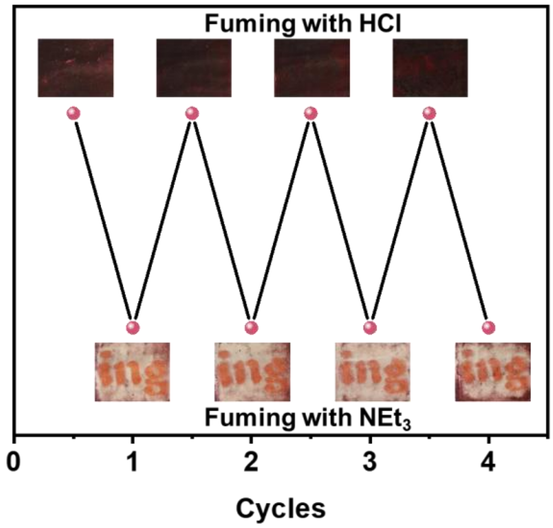

Supplementary Fig. 34 | Repeatability test. The disappearance and reappearance of information during four base-acid cycles.

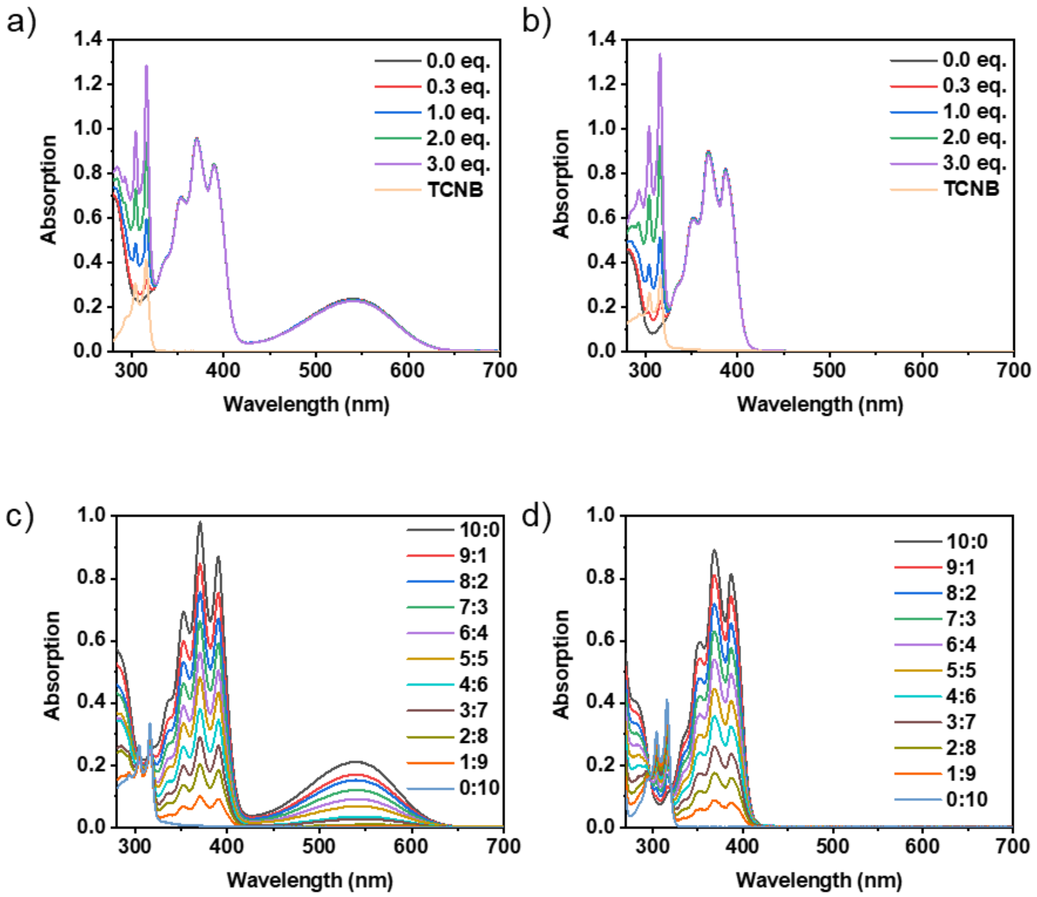

Supplementary Fig. 35 | UV-visible titrations of AIO and AIC solutions with TCNB. a) UV-vis spectra of AIO in acetonitrile solution (0.1 mM) with varying amounts of TCNB (0–3.0 equiv.). b) UV-vis spectra of AIC in acetonitrile solution (0.1 mM) with varying amounts of TCNB (0–3.0 equiv.). c) UV-vis spectra of the AIO/TCNB complex formed by mixing different molar ratios of AIO and TCNB in acetonitrile solution (0.1 mM). d) UV-vis spectra of the AIC/TCNB complex formed by mixing different molar ratios of AIC and TCNB in acetonitrile solution (0.1 mM). The UV-visible spectra showed minimal changes, indicating weak CT interactions in solution (Fig. S35a and S35b). Due to the weak nature of these interactions, Job's plot method could not reliably determine the binding ratio between AIO/AIC and TCNB in solution (Fig. S35c and S35d).

Section D. Characterization of new compounds

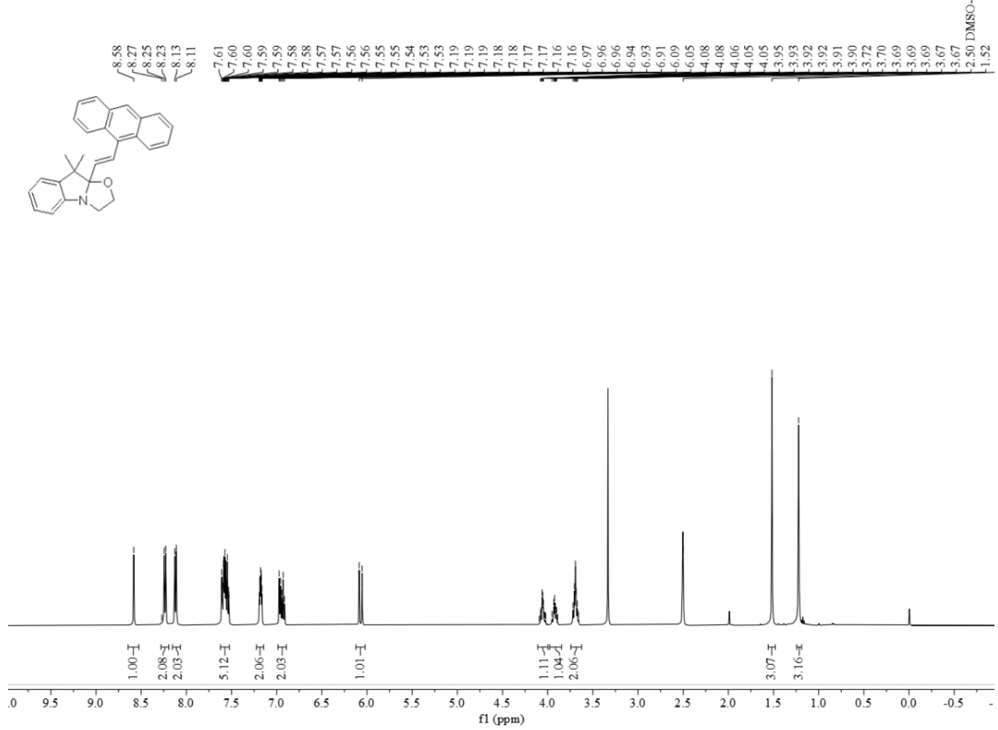

Supplementary Fig. 36 | ^{1}H NMR analysis. ^{1}H NMR spectrum of AIC (DMSO-d_6, 500 MHz).

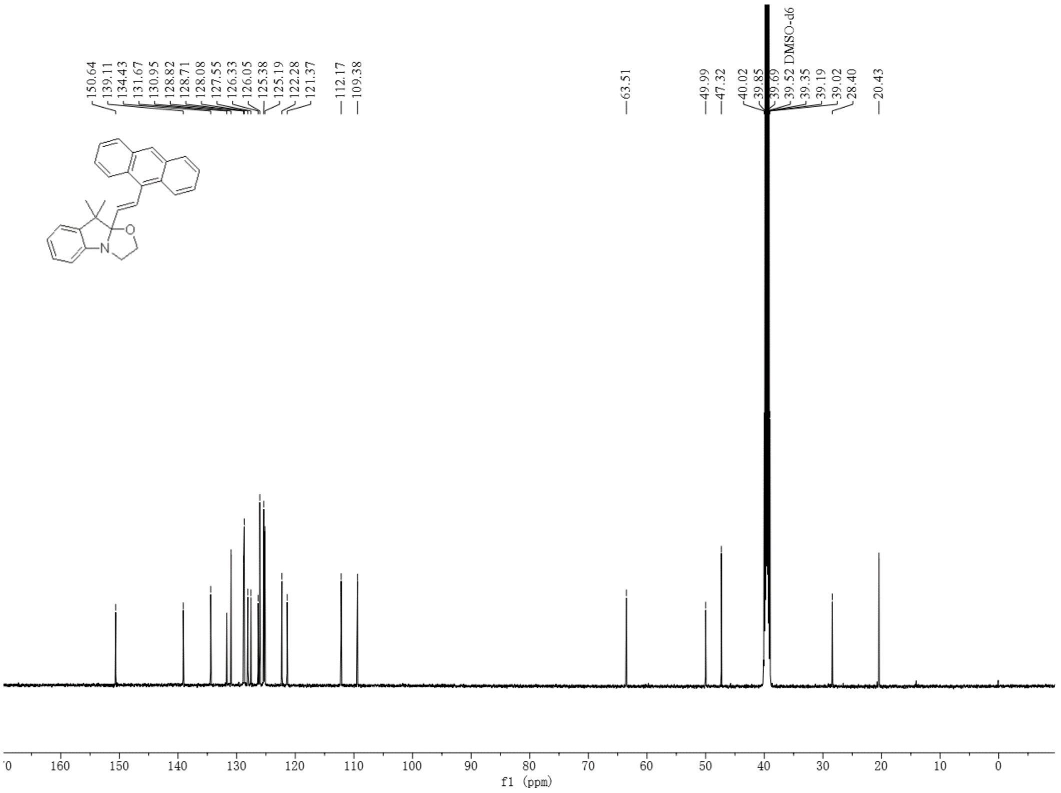

Supplementary Fig. 37 | ^{13}C NMR analysis. ^{13}C NMR spectrum of AIC (DMSO-d_6, 125 MHz).

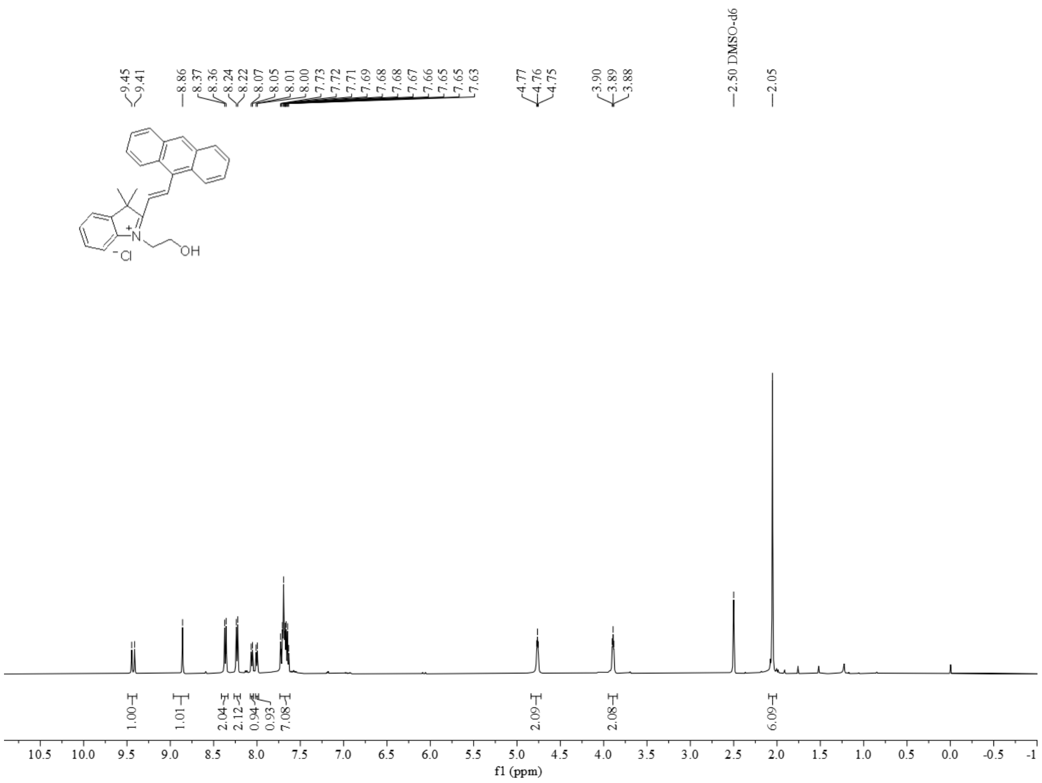

Supplementary Fig. 38 | ^{1}H NMR analysis. ^{1}H NMR spectrum of AIO (DMSO-d_6, 500 MHz).

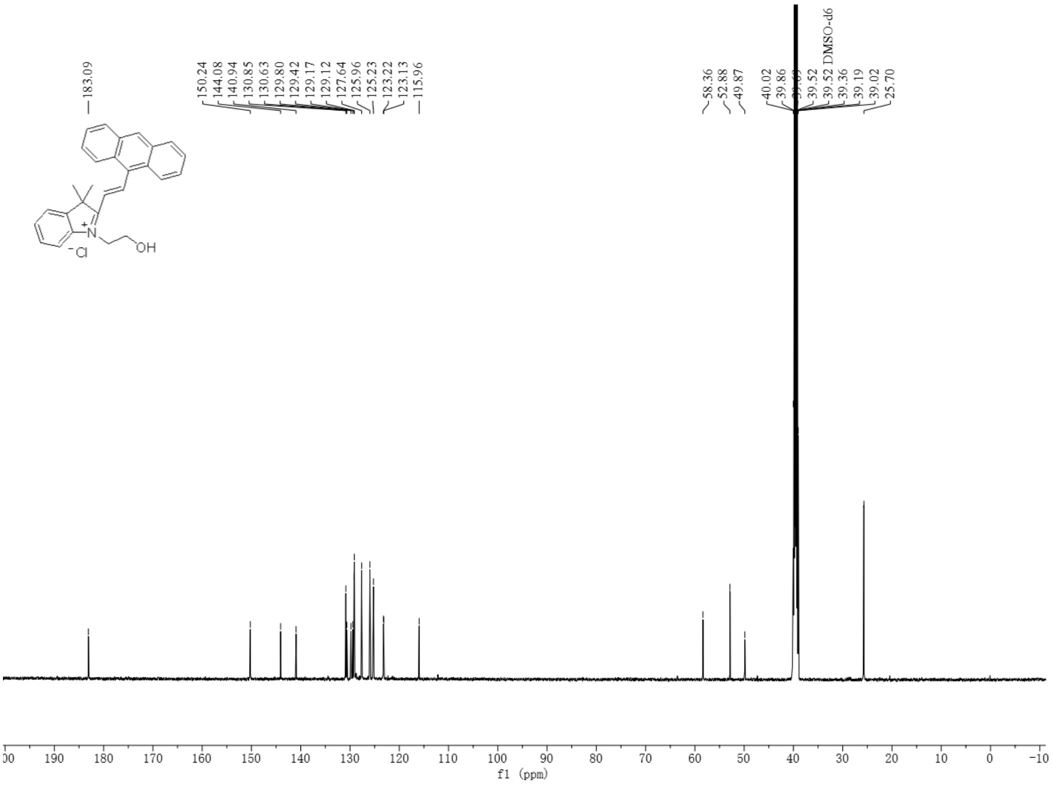

Supplementary Fig. 39 | ^{13}C NMR analysis. ^{13}C NMR spectrum of AIO (DMSO-d_6, 125 MHz).

Section E. Supplementary X-ray crystallographic data.

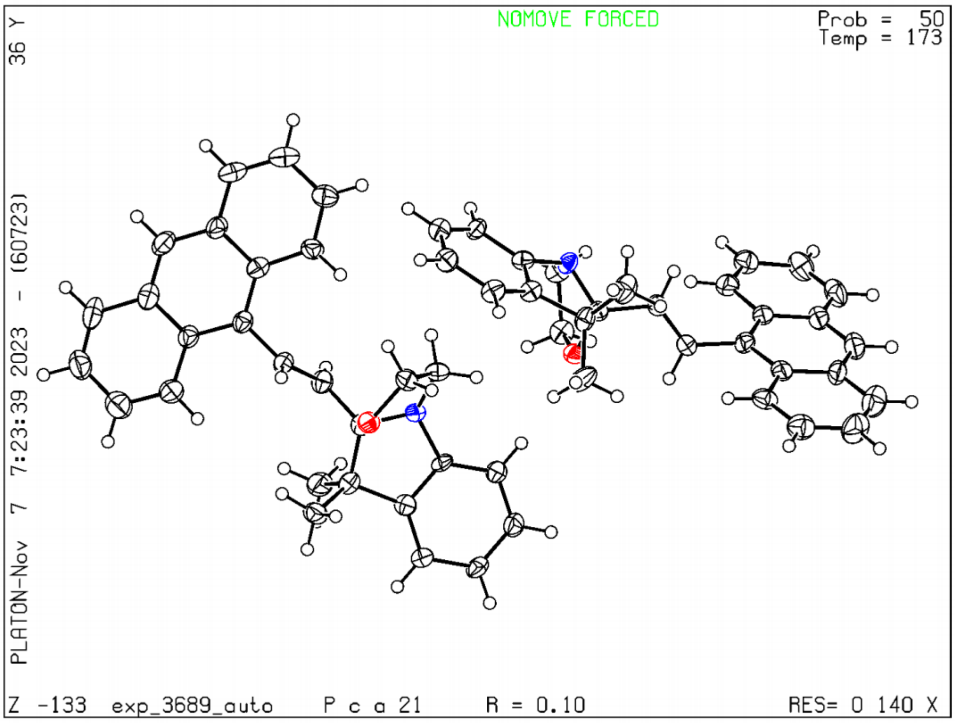

Supplementary Fig. 40 | X-ray crystallographic data. X-ray crystallographic data for AIC (CCDC: 2343124). One B-Level alert for the AIC crystal remains unresolved due to crystal twinning. Despite several attempts to resolve this issue, this is currently the best available data.

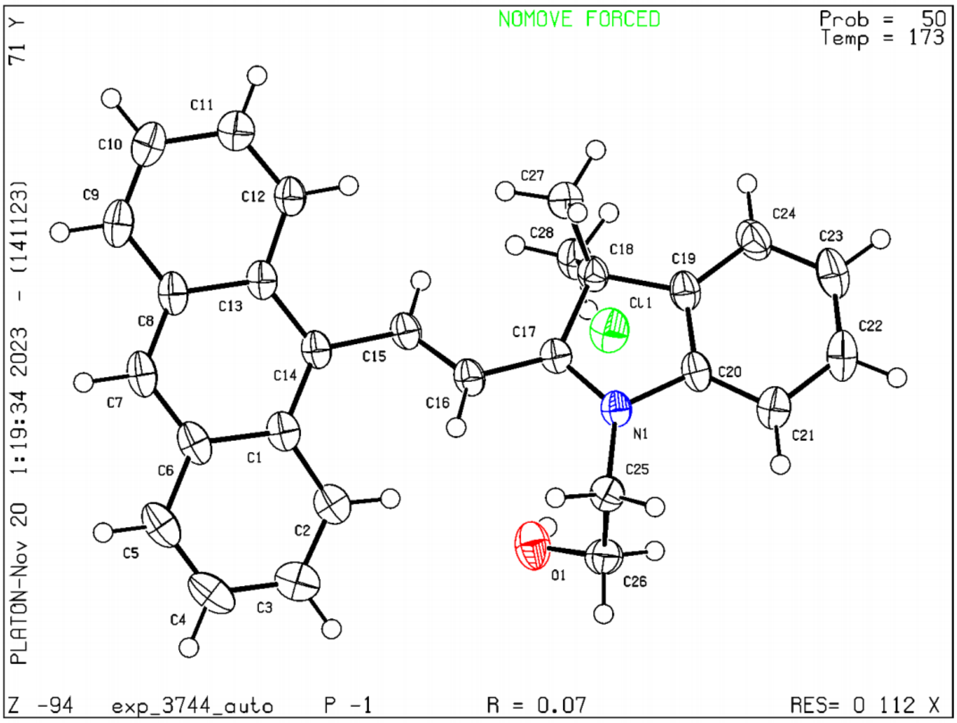

Supplementary Fig. 41 | X-ray crystallographic data. X-ray crystallographic data for AIO (CCDC: 2343125).

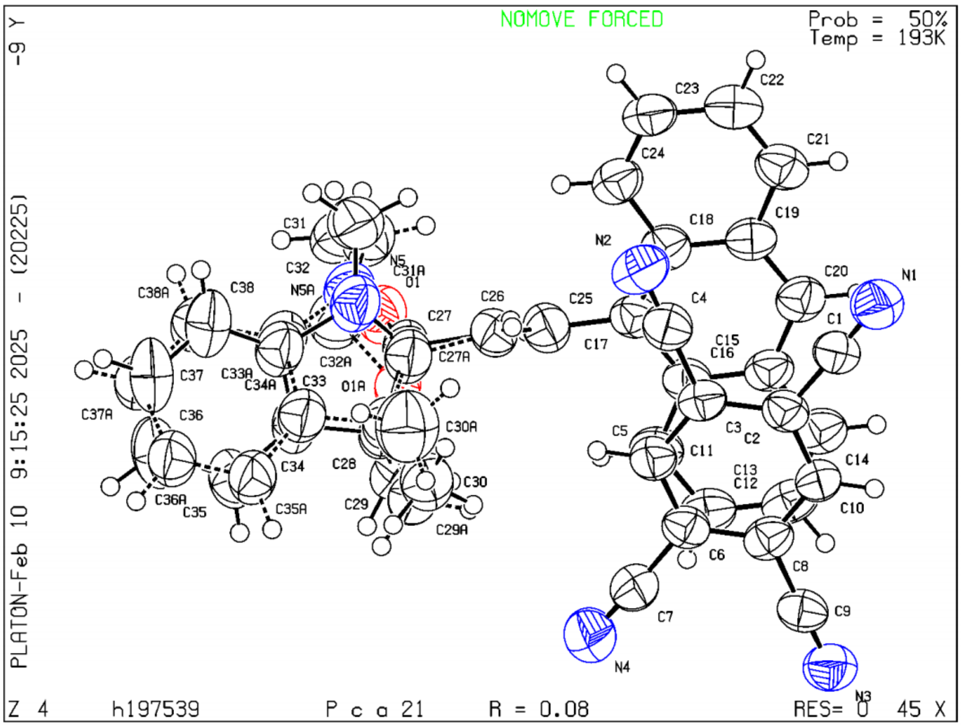

Supplementary Fig. 42 | X-ray crystallographic data. X-ray crystallographic data for ACTC (CCDC: 2343126).

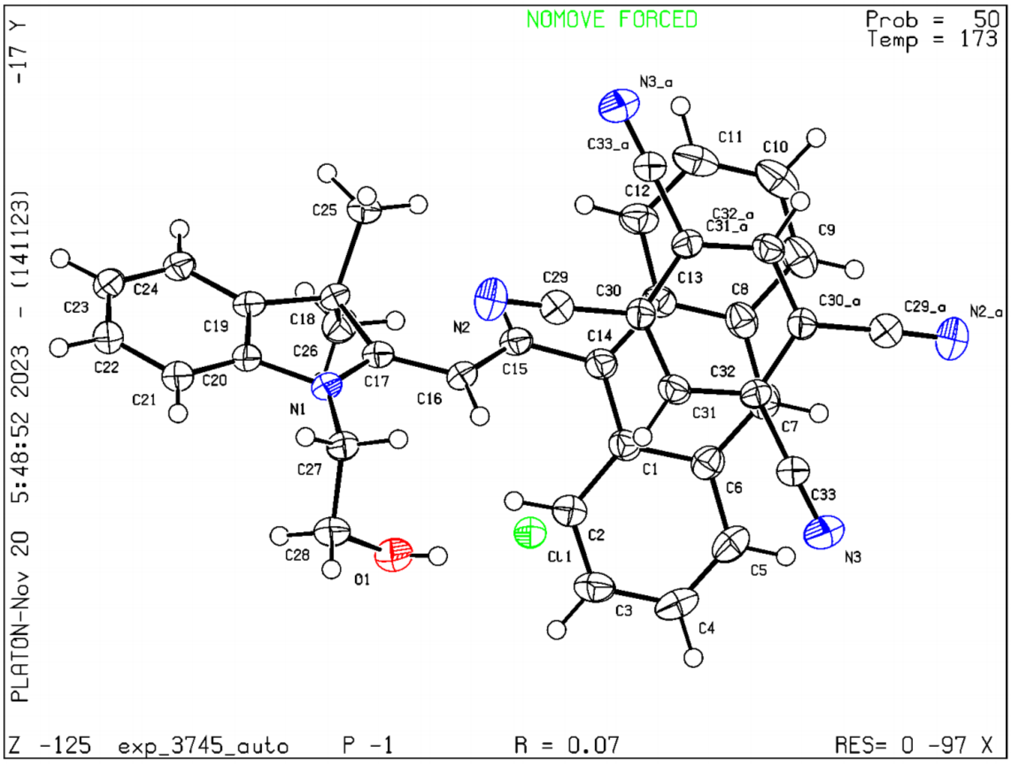

Supplementary Fig. 43 | X-ray crystallographic data. X-ray crystallographic data for AOTC (CCDC: 2343127).

Reference

1. Frisch, M. J. et al. Gaussian, Inc., Wallingford CT, 2016.
2. Yanai, T., Tew, D. P. & Handy, N. C. A new hybrid exchange–correlation functional using the Coulomb-attenuating method (CAM-B3LYP). *Chem. Phys. Lett.* **393**, 51-57 (2004).
3. Grimme, S., Antony, J., Ehrlich, S. & Krieg, H. A consistent and accurate ab initio parametrization of density functional dispersion correction (DFT-D) for the 94 elements H-Pu. *J. Chem. Phys.* **132**, 154104 (2010).
4. Grimme, S., Ehrlich, S. & Goerigk, L. Effect of the damping function in dispersion corrected density functional theory. *J. Comput. Chem.* **32**, 1456-1465 (2011).
5. Martin, R. L., Hay, P. J. & Pratt, L. R. Hydrolysis of Ferric Ion in Water and Conformational Equilibrium. *J. Phys. Chem. A* **102**, 3565-3573 (1998).
6. Qu, S. et al. Catalytic Mechanisms of Direct Pyrrole Synthesis via Dehydrogenative Coupling Mediated by PNP-Ir or PNN-Ru Pincer Complexes: Crucial Role of Proton-Transfer Shuttles in the PNP-Ir System. *J. Am. Chem. Soc.* **136**, 4974-4991 (2014).
7. Wang, Y., Ling, B., Liu, P. & Bi, S. A Reaction Mechanism for Gold-Catalyzed Hydroamination/Cyclization of o-Phenylendiamine and Propargylic Alcohols. A DFT Study. *Organometallics* **37**, 3035-3044 (2018).
8. CYLview20; Legault, C. Y., Université de Sherbrooke, **2020** (http://www.cylview.org).
9. Lu, T. & Chen, F. Multiwfn: A multifunctional wavefunction analyzer. *J. Comput. Chem.* **33**, 580-592 (2012).
10. Humphrey, W., Dalke, A. & Schulten, K. VMD: Visual molecular dynamics. *J. Mol. Graphics* **14**, 33-38 (1996).
